# Recent biomedical advances enabled by HaloTag technology

**Published:** 2022-04-22

**Authors:** Weiyu CHEN, Muhsin H. YOUNIS, Zhongkuo ZHAO, Weibo CAI

**Affiliations:** 1 The Fourth Affiliated Hospital, Zhejiang University School of Medicine, Yiwu, 322000, China; 2 International Institutes of Medicine, The Fourth Affiliated Hospital of Zhejiang University School of Medicine, Yiwu, 322000, China; 3 Departments of Radiology and Medical Physics, University of Wisconsin—Madison, Madison, WI, 53705, USA

**Keywords:** HaloTag, Site-specific labeling, Biomolecule interaction, Molecular imaging, Positron emission tomography

## Abstract

The knowledge of interactions among functional proteins helps researchers understand disease mechanisms and design potential strategies for treatment. As a general approach, the fluorescent and affinity tags were employed for exploring this field by labeling the Protein of Interest (POI). However, the autofluorescence and weak binding strength significantly reduce the accuracy and specificity of these tags. Conversely, HaloTag, a novel self-labeling enzyme (SLE) tag, could quickly form a covalent bond with its ligand, enabling fast and specific labeling of POI. These desirable features greatly increase the accuracy and specificity, making the HaloTag a valuable system for various applications ranging from imaging to immobilization of POI. Notably, the HaloTag technique has already been successfully employed in a series of studies with excellent efficiency. In this review, we summarize the development of HaloTag and recent advanced investigations associated with HaloTag, including *in vitro* imaging (e.g., POI imaging, cellular condition monitoring, microorganism imaging, system development), *in vivo* imaging, biomolecule immobilization (e.g., POI collection, protein/nuclear acid interaction and protein structure analysis), targeted degradation (e.g., L-AdPROM), and more. We also present a systematic discussion regarding the future direction and challenges of the HaloTag technique.

## Introduction

A series of biological processes (e.g., development of diseases) are kept happening in the body, which was triggered by complex interactions between biomacromolecule (e.g., protein-protein or protein-nucleic acid). Therefore, a comprehensive understanding of these interactions would reveal the mechanisms of certain diseases and advance the strategies for early diagnoses and therapies. Given that cellular proteins are highly dynamic, genetic modification is the best approach for investigating a protein of interest (POI) at active status. Generally, the labeling of POI via affinity tag (e.g., His-Tag) or fluorescent protein (e.g., GFP) allows an intracellular tracking or immobilization of POI. However, several drawbacks cannot be avoided entirely. For instance, the impurity of harvested proteins could be caused by unspecific binding (e.g., His-Tag) or a decrease in the brightness of fluorescence, etc. These disadvantages potentially restrain their applications in studies that require high accuracy (e.g., miRNA-protein interaction or POI tracking studies).

**SCHEME 1.**
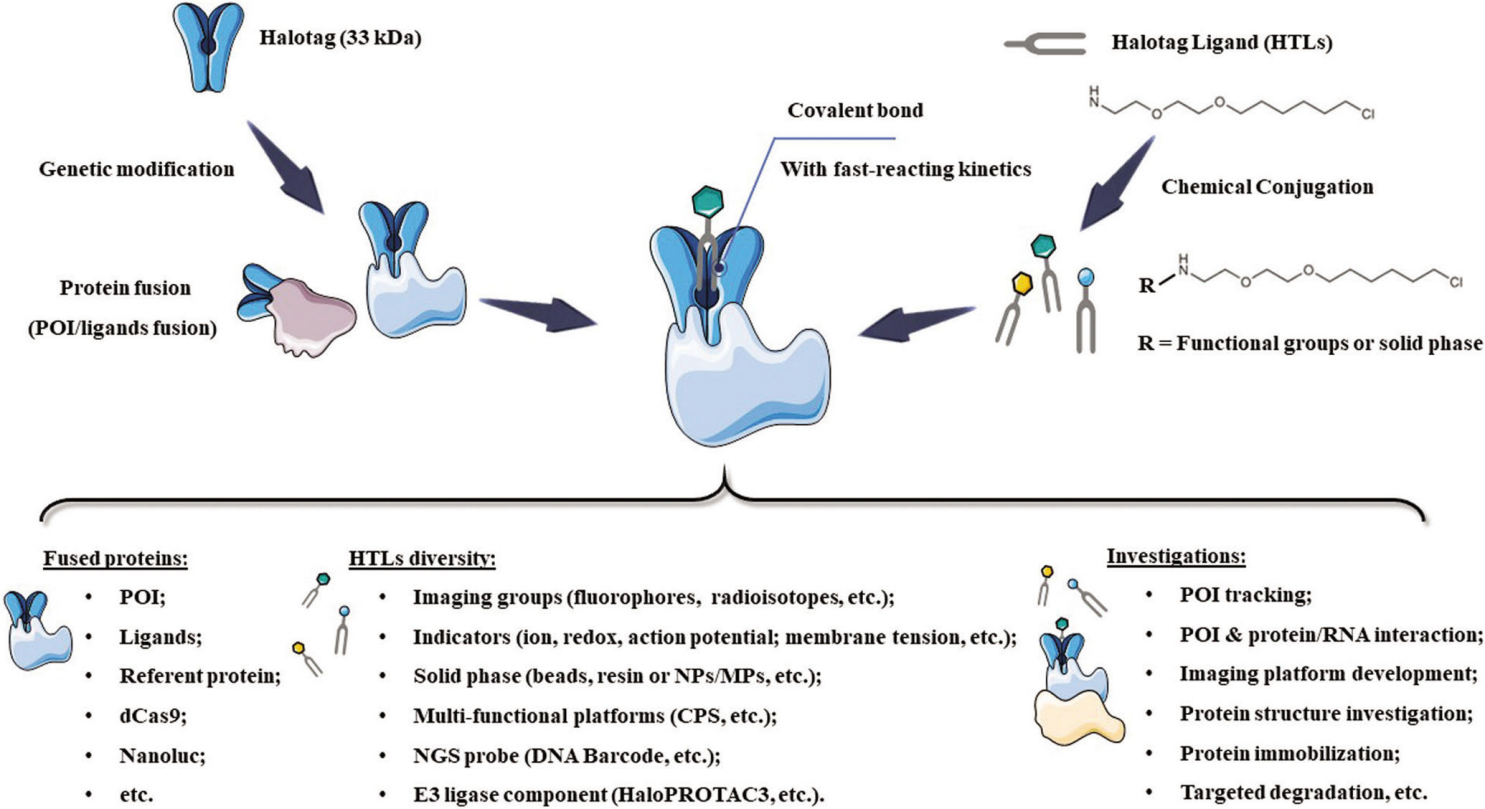
Scheme of HaloTag system, as well as the recent designs and investigations. NPs: nanoparticles; MPs: micro-sized particles; NGS: Next-Generation Sequencing; POI: Protein of Interest; CPS: Cell-penetrating streptavidin.

In the last two decades, a series of self-labeling enzymes, also called self-labeling enzymes (SLEs), had been successfully developed. Three prominent examples are the CLIP-tag/SNAP-tag (19.4 kDa), ACPtag (9 kDa) and HaloTag (33 kDa) (Table 1). These SLEs share several features, including relatively small size, high stability, and fast-reacting kinetics, allowing fused POI to be labeled by tag-specific substrates with extremely high specificity and efficiency. More importantly, the functionalization of interactive substrates allows fusion proteins to be immobilized on a solid phase, monitored via molecular imaging (e.g., fluorescence, luminescence or radioactive isotope, etc.) or gifted capacities (e.g., redox-response or ion-activated imaging). As such, the POI or the interacting biomolecules on the POI could be specifically studied via the functional substrates that covalently bind to POI for various purposes, even high-resolution imaging of cellular ion influx. In the current review, we summarize the developments of HaloTag technology, in particular, recent applications of HaloTag for POI tracking (e.g., *in vitro* and *in vivo* imaging), immobilization (e.g., protein-reacting mRNA analysis and protein structure study), targeted degradation (e.g., chloroalkane-containing proteolysis targeting chimeric (HaloPROTACs)) and other applications (e.g., differentiation of stem cell), is highlighted (Scheme 1). In addition, the prospects and challenges for future developments of HaloTag are systemically discussed.

## A Brief History of HaloTag Technology

HaloTag (33 kDa) is modified from the bacterial enzyme haloalkane dehalogenase, with its Phe272 residue mutated to His272. The mutation of this residue results in a loss of its ability to perform hydrolysis. As a result, an alkyl-enzyme intermediate can be formed between the HaloTag and its ligand without further hydrolysis, leading to the formation of a covalent bond. Although HaloTag is an exogenous protein, it does not interfere with an organism’s normal physiological metabolism (Naested et al., 1999). By recruiting different designs of ligands, HaloTag is applicable in various directions of research and investigation. For example, the folding of HaloTag can be directly monitored via the change of fluorescent intensity from a conjugated ligand, which may serve as a desirable platform to study the POI folding procedures during translation or other high-complexity conditions (Samelson et al., 2018).

Since Promega Corporation developed HaloTag in 2005 (Los et al., 2005), this technique has received growing attention from scientists worldwide. Within one year, the high efficiency of HaloTag system was investigated and verified by several research groups (Lang et al., 2006; Zhang et al., 2006). For instance, the Rao group successfully used HaloTag technology to induce a site-specific modification of bioluminescent protein on quantum dots (QDs) (Zhang et al., 2006). As-prepared QDs were labeled with HaloTag-fused renilla luciferase fusion protein via surface conjugation of HaloTag ligand (HTL), which could be lightened up by bioluminescence resonance energy transfer (BRET) after the attendance of the substrate, coelenterazine. Although HaloTag was only designed for applications in mammalian cells initially, researchers ingeniously employed this platform in a series of different research fields. In the following decade, HaloTag technique was comprehensively studied and applied in studies including protein interactions (Löchte et al., 2014), localization of POI within cells (Lee et al., 2010; Strauch et al., 2011; Liu et al., 2012), and functional HaloTag ligand (HTL) developments (Raina et al., 2014; Neklesa et al., 2011). In particular, advanced HTL such as HyT36 and HyT13 generated from the Crews group has paved new ways of observing certain mechanisms (e.g., endoplasmic reticulum (ER) regulation) inside cells (Raina et al., 2014; Neklesa et al., 2011). More specifically, these HTL could induce the destabilization of HaloTag-fused POI on ER and cause a resolvable ER stress, revealing the relation between unfolded protein response (UPR) and estrogen-mediated ER stress. With these fundamental research bases, HaloTag gradually became a powerful tool that has been widely applied in various basic and applied researches (Schlichthaerle et al., 2019; Li et al., 2020). In the following sections, we will summarize these latest and novel investigations associated with HaloTag strategy (Table 2).

## Recent Biomedical Applications of HaloTag Technology

### In vitro imaging

Imaging POI in living or fixed cells could offer vital information for understanding their biochemical functions inside protein networks (Ohno et al., 2014). In support of HaloTag system, POI fused with HaloTag could be effectively labeled by HTL. As such, a series of approaches for tracking POI in cell or micro-organs, monitoring cellular conditions and developing advanced platforms for therapy or imaging could be achieved via different conjugations of functional groups on HTL, such as dyes (e.g., rhodamines, carbopyronines, Si-rhodamine, Alexa Fluor) (Takahashi et al., 2019; Frei et al., 2019; López-Andarias et al., 2020; Thevathasan et al., 2019).

### POI imaging

**FIGURE 1.**
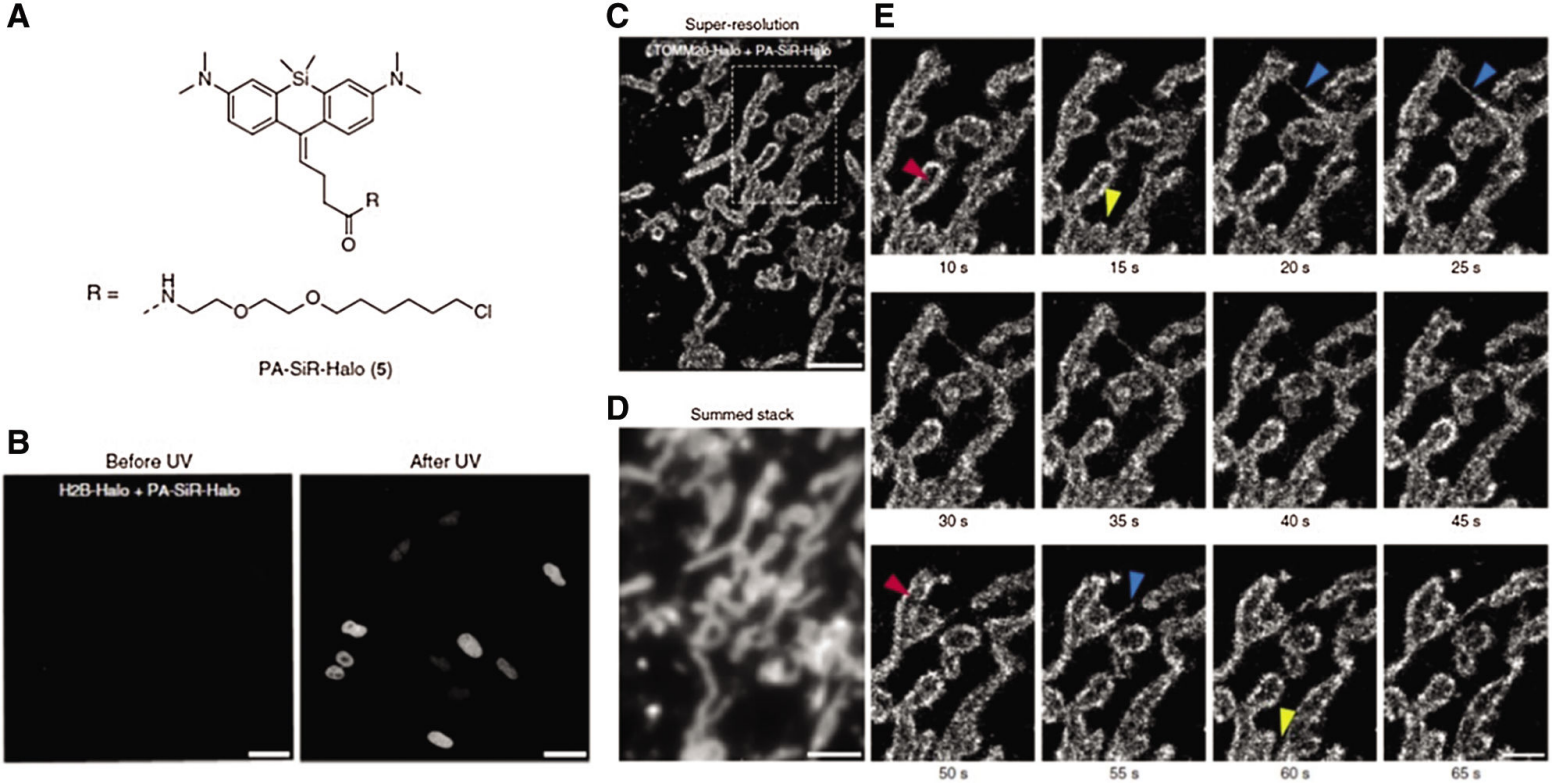
(A) Chemical structure of PA-SiR-Halo. (B) Living cell imaging on U-2 OS cells expressing H2B-Halo (nucleus) stained with PA-SiR-Halo before and after UV irradiation via SMLM; Scale bar, 40 μm. (C) Super-resolved SMLM image of outer mitochondrial membrane (TOMM20-Halo) after PA-SiR-Halo staining. (D) Summed stack image for mimicking diffraction-limited image. (E) Series of images at different time points. Arrowheads indicate hollow mitochondria (due to the TOMM20 localized to the outer membrane) (red), thin tubules formed by highly dynamic mitochondria neighboring mitochondria (blue) and disconnect (fission) in other areas (yellow); Scale bar, 1 μm. Reproduced with permission from Frei et al. (2019).

As the general approach for POI imaging, HaloTag is directly fused with POI (e.g., G Protein-coupled receptors and receptor tyrosine kinases, etc.), enabling site-specific labeling via HTL and imaging groups conjugated (e.g., fluorophores) (Berki et al., 2019; Butkevich et al., 2016; Lesiak et al., 2020; Peach et al., 2021). For instance, a HaloTag fused serum response factor (SRF) was designed by Hipp et al. (2019) to investigate the interaction between SRF and chromatin in fibroblast and primary neuron cells. Significantly, the fusion of HaloTag did not induce any interference on SRF’s location and functions. With the assistance of HTL-fluorophore (TMR and silicone rhodamine), chromatin residence times of SRF could be accurately detected (up to 1 min) and defined as three regimes via single-molecule living imaging. Similarly, Frei et al. (2019) successfully visualized HaloTag-fused POI (TOMM20) on the outer mitochondrial membrane via single-molecule localization microscopy (SMLM) (Fig. 1A). Specifically, novel photoactivatable silicon rhodamine (PA-SiR) with super spectroscopic properties was developed and conjugated on HTL. Under UV irradiation (Fig. 1B), PA-SiR bound to TOMM20 could be protonated and visualized via SMLM for over 65 s in living cells (Figs. 1C-1E). In addition, Huet-Calderwood et al. (2017) reported a complex platform for imaging integrin *in vitro* by integrating various fluorescent tags (e.g., GFP, pHluorin, or HaloTag) with β1 integrin (ecto-β1 via HaloTag technique. In particular, the highly specifical ecto-Halo β1 was able to track and analyze β1 integrin with detailed spatiotemporal dissection for up to 1 h, showing the high accuracy of HaloTag strategy.

**FIGURE 2.**
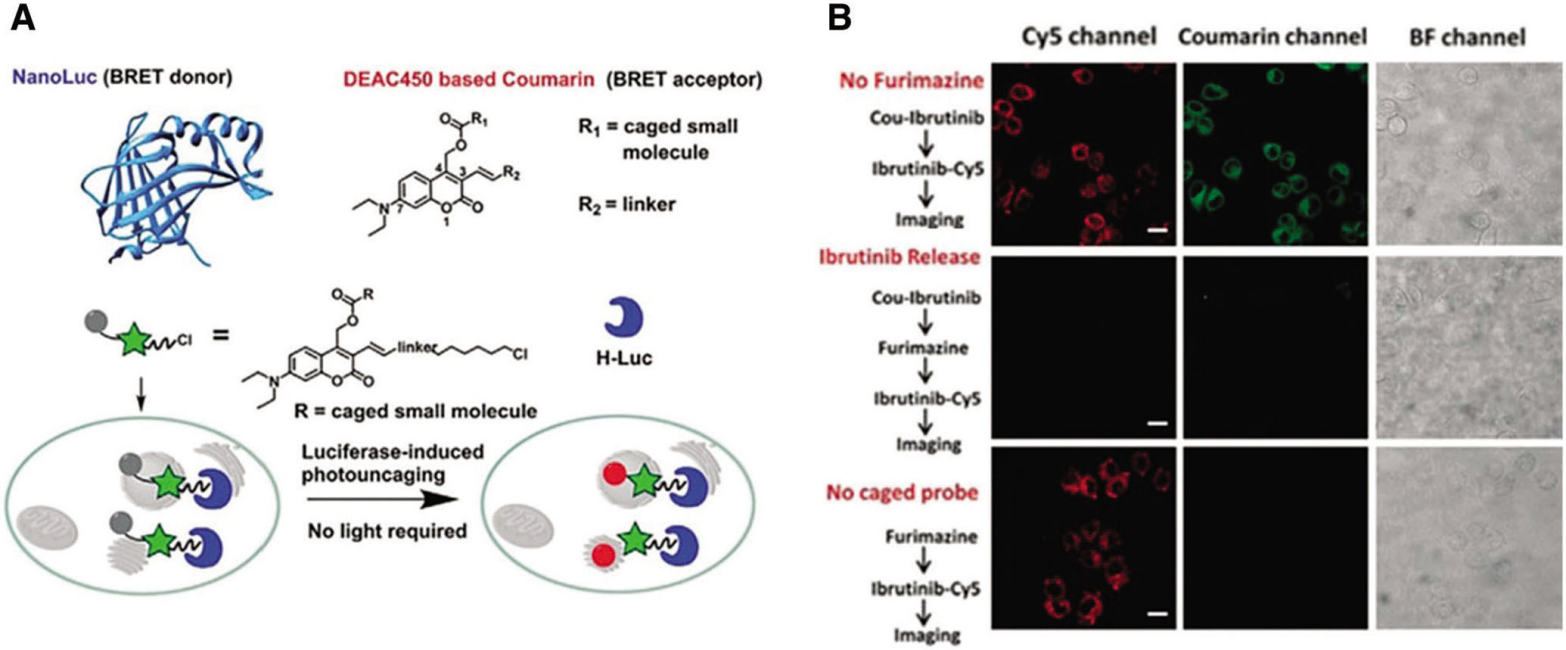
(A) Scheme of BRET-induced photolysis to release small molecule, bioluminolysis. Briefly, the BRET will happen between nanoluc (fused with HaloTag) and DEAC450 (conjugated with HTLs) when furimazine encounters with nanoluc. (B) The release of ibrutinib mediated by coumarin BRET photo-uncaging in live SKBR3 cell. Scale bar = 20 mm. Reproduced with permission from Chang et al. (2019).

Unlike most studies recruiting conventional fluorophores, a light-independent/luminescent imaging strategy was developed by Chang et al. (2019) (Figs. 2A and 2B). In this system, HaloTag was fused with nanoluc that could behave as a bioluminescence resonance energy transfer (BRET) donor. Once the furimazine was presented, nanoluc will be lighted up and excite HTL-DEAC450 (coumarin-based dye), with fast kinetics (t_1/2_ < 120 s) (Fig. 2A). More importantly, after photolysis, the uncaging ibrutinib from HTL-DEAC450 induced efficient therapy on HeLa and SKBR3 cells (Fig. 2B).

However, the imaging of single POI only provides limited information, which is insufficient for studying interactions between biomacromolecules or high-accuracy researches. Thus, innovative dual-tracking approaches have been developed for identifying the interactions between POI & POI or POI & RNA (Sato et al., 2017; Yoon et al., 2016). For example, the Singer group introduced a modular design for tracking a POI and its mRNA simultaneously in neurons from genetic-modified mice. The β-actin mRNA was encoded with 24 tandem MS2 aptamers at 3’-UTR, while HaloTag was fused to tag β-actin. With the attendance of endogenous-expressed stdMCP-stdGFP (MS2 capsid protein (MCP) and GFP fusion protein) and fluorescent HTL (i.e., JF549/JF646), β-actin mRNA and β-actin could be visualized simultaneously with high resolution.

Instead of dual-labeling, incorporating multiple HTL (with different physiochemical features) and HaloTag-fused POI could also help some investigations on biological progress. As one representative application, Takahashi et al. (2018, 2019) tactfully utilized HaloTag-fused microtubule-associated protein one light chain 3 (HT-LC3) and different HTL (cell-penetrated/unpenetrated) to visualize the procedure of autophagosome formation. In addition, Takahashi *et al*. also recruited HaloTag system for investigating endosomal sorting complexes that were required in the formation of transport-III (ESCRT-III) component (CHMP2A) and ESCRT-I subunit (VPS37A). The genome screen on CRISPR library and HT-LCs platform successfully identify these complexes as critical factors for phagophore completion.

### Monitoring cellular status

Physiochemical conditions are of vital importance for maintaining cellular functions and responses. Using HaloTag, investigators have developed a series of site-specific sensors for detecting the alteration of ion level (e.g., Mg^2+^, Na^+^, Zn^2+^, Ca^2+^) (Deo et al., 2019; Gruskos et al., 2016; Matsui et al., 2017; Taguchi et al., 2018; Zastrow et al., 2020), redox change (Jiang et al., 2019; Parvez et al., 2016), action potential (Jiang et al., 2019; Parvez et al., 2016) and cell membrane tension (Strakova et al., 2020). In consideration that sodium and calcium ions are two crucial secondary messengers associated with most biochemical activities, Deo et al. (2019) synthesized novel Ca^2+^ indicators that were conjugated with HTLs. Among a series of HTL-indicators, the 12_AM_ synthesized could generate a site-specific and bright signal that was approximately 7.8 times that produced by jRGECO1a (verified Ca^+^ indicator) in hippocampal neurons. More importantly, the Ca^2+^ influx of an entire organelle (primary cilium, HaloTag-fused 5HT_6_) was clearly visualized by the HTL-indicator (13_AM_) after stimulation of o-nitrophenyl-EGTAAM.

**FIGURE 3.**
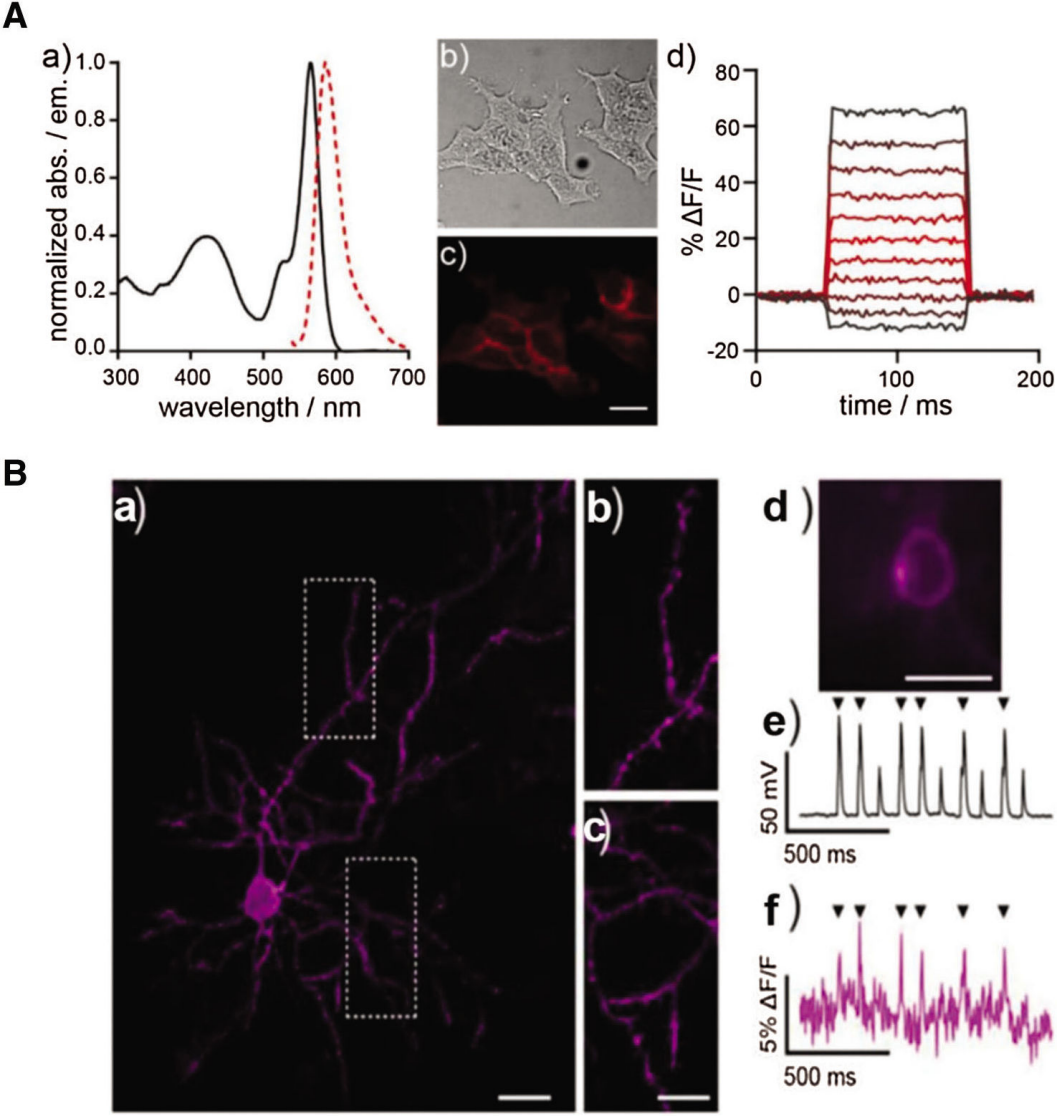
(A) The characterization of voltage sensor RhoVR. (a) Normalized spectra of absorption and emission at the concentrate of 500 nM; (b) Blight field and (c) fluorescence imaging of HEK293T cells with staining of RhoVR. (d) Plot of fractional change in terms of fluorescence (ΔF/F) *vs.* time; (B) Imaging of brain slice isolated from a mouse expressing pDisplay-HaloTag via two-photon microscopy after staining of RhoVR-Halos. (a) RhoVR fluorescence imaging of brain slide and (b) & (c) enlarged images; Scale bar is 10 μm. (d) Widefield fluorescence image of RhoVR-Halos fluorescence in a cortical neuron expressing HaloTag-pDisplay; Scale bar is 20 μm. (e) Plot of voltage *vs.* time for the neuron during current injection to evoke action potentials. (f) Plot of ΔF/F *vs.* time for the same neuron. Arrows indicate evoked spike. Reproduced with permission from Deal et al. (2020).

Although action potential is strongly related to calcium ion influx, the kinetics of voltage gating is too fast to be monitored by general Ca^2+^ indicators. Inspired by photo-induced electron transfer (PeT), Dear et al. (2020) reported voltage-sensitive rhodamine (RhoVR-Halos) that was conjugated to HTL with polyethyleneglycol (PEG) as the linker (Figs. 3A and 3B). RhoVR-Halos effectively bound to HaloTag-fused membrane (Fig. 3A), showing high sensitivities in HEK cells (34% ± 2% ΔF/F per 100 mV), cultured rat neurons (6.7% ± 0.2% ΔF/F per spike) and neurons in slice (4.3% ± 0.3% per spike). Furthermore, a dual-functional indicator consisting of RhoVR-Halos and GCaMP (Ca^2+^ indicator) was successfully developed to monitor V_m_ and Ca^2+^ influx simultaneously (Fig. 3B).

Given the crucial role of redox in cellular homeostasis, several sensors have been investigated for surveying redox status (Jiang et al., 2019; Parvez et al., 2016). Parvez et al. (2016) creatively established a redox-monitoring technique named Targetable Reactive Electrophiles and Oxidants (T-REX) by recruiting a lipid-derived signaling electrophiles endogenous carrier (4-hydroxynonenal (HNE)) and HaloTag platform. This universal platform was capable of monitoring dynamic redox change around HaloTag-labeled POI by photo-mediated uncaging (t_1/2_ < 1–2 min) (HTL-PreHNE), exhibiting potential as a powerful tool for screening redox-related targets. At the same time, Jiang et al. (2019) succeeded in synthesizing a smart HTL-RealThiol (HLT-RT) that could achieve a site-specific GSH detection (nucleus and cytosol). Specifically, HLT-RT was able to detect GSH in HeLa cells and primary hepatocytes via ratiometric fluorescence (blue/green channel fluorescence). The employment of this advanced reversible probe (HTL-RT) in T-REX may efficiently prolong the timespan for organelle or cell imaging, achieving a lifetime survey in a real-time manner.

Interestingly, Strakova et al. (2020) developed a sensor to probe membrane tension, which is now a commercial product, Flipper-TR. This sensor functions by generating red-shift fluorescence and extending its lifetime by changing “twist” form to “planar” structure. Once the tagged cell membrane became tense, the signal could be precisely excited and the change of lifetime was recorded, with an increase of about 0.37 to 0.27 ns and −0.02 ns for rhodamine (control).

### Imaging of microorganisms

Similarly, a comprehensive understanding of microbes would effectively promote the development of interrelated diagnosis and therapy as well. In recent years, several HaloTag-assisted studies have been reported for investigating microbes, including bacterial imaging (Barlag et al., 2016; Lepore et al., 2019; Spencer et al., 2018), evaluation of therapeutic agents (Yang and Weisshaar, 2018), and virus tracking (Liu et al., 2016). As an illustration, Liu et al. (2016) used HaloTag to label VP26, the smallest capsid protein on the pseudorabies virus (PrV). Eventually, they succeeded in visualizing two generations (parental and progeny) of PrV inside cells with HTLs (TMR and R110), which offers valuable information for understanding the whole lifespan of PrV inside cells. Additionally, Yang and Weisshaar (2018) created genetically engineered *E. coli* expressing HaloTag in outer and cytoplasmic membranes (OM/CM). This model was subsequently used to evaluate therapeutic effects of anti-bacterial agents (MM63:CHx37, AMP LL-37, and AMP CM15) according to the permeabilization of HTL-JF646 in treated *E. coli.*

### Imaging/delivery system development

The desirable specificity of HaloTag efficiently ensures its accuracy and stability, potentially extending its application for developing novel imaging/delivery systems, such as enabling specific POI as the high-fidelity control for advanced microscope development. Similar to the previously described technique for anti-bacterial evaluation, several assessment platforms based on HaloTag technique were reported too. For example, the chloroalkane penetration assay (CAPA) was designed as a high-throughput assessment for evaluating the cell penetration of therapeutic agents under various conditions (e.g., variations in temperature, time and serum), assisted by HTL and an organelle-specific organelle-specific HaloTag (Peraro et al., 2018).

**FIGURE 4.**
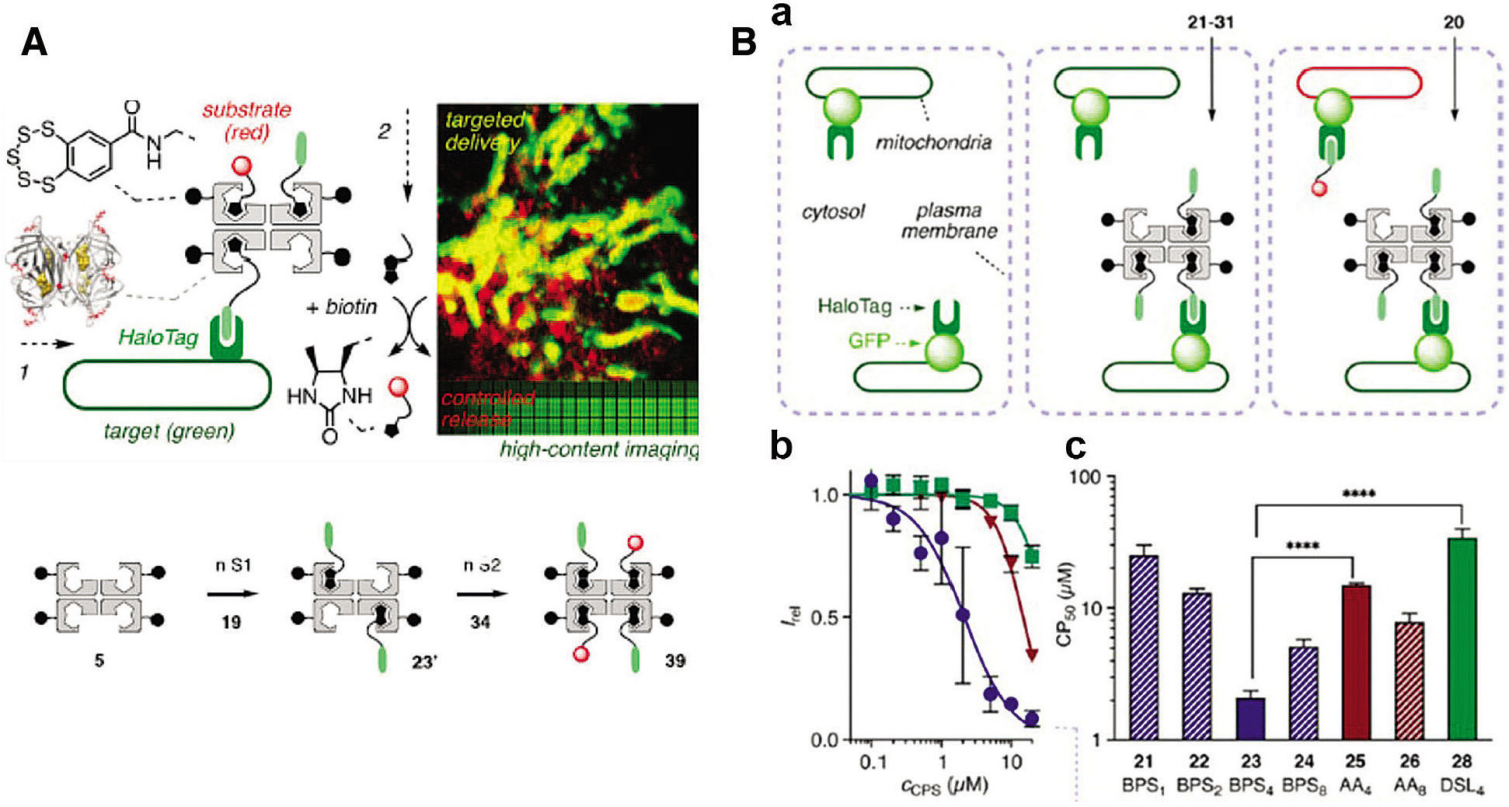
(A) Scheme of cell-penetrating streptavidin (CPS) system. The CPS is able to load different functional groups via streptavidin and biotin interaction, which could be integrated with HaloTag technique for site-specific drug delivery or imaging. (B) (a) Schematic representation of CAPA. (b) HC-CAPA dose–response curves for different CPS loading cargos, 23 (Circle), 25 (Triangle), and 28 (Square). (c) The CP_50_ values of various CPS loading cargos 21–26 and 28. Reproduced with permission from López-Andarias et al. (2020).

It is notable that a creative streptavidin-based platform and a series of small molecules have been synthesized for multiple applications (López-Andarias et al., 2020). This chemical group, cell-penetrating streptavidin (CPS), consists of 4 binding sites for loading or releasing cargos via desthiobiotin/biotin interaction or desthiobiotin-biotin exchange (Fig. 4A). With the support of chloroalkane penetration assay (CAPA) and HaloTag system, López-Andarias et al. (2020) were able to screen the best combination (CPS carrying four benzopolysulfanes, BPS_4_) for cytosolic delivery from numerous CPS-loaded drug candidates, potentially indicating the high efficiency of HaloTag technology (Fig. 4B).

Meanwhile, HaloTag has been involved in the microscope system or related dye developments as well. The method for single-molecule localization microscopy (SLSM, also named photoactivated localization microscopy, PALM) calibration is remarkably restrained due to the insufficient control data. To remedy this, Thevathasan et al. (2019) used genetic modification to produce various Nup96 proteins, the major component in nucleic pore complex (NPC) fused with a series of tags (e.g., HaloTag). With high-resolution imaging quality and super-fidelity supported by HaloTag, these engineering cells were successfully used as references for SLSM development, including resolution calibration, labeling efficiencies quantification, and molecular counting. In the same year, an innovative imaging strategy for DNA point accumulation in nanoscale topography (DNA-PAINT) was also developed (Schlichthaerle et al., 2019). Schlichthaerle *et al.* used dye-labeled docking DNA to induce transient labeling on Nup96-Halo-HLT-DNA. These strategies resulted in desirable SLSM images with excellent resolution. Such measured distance on adjacent Y-complexes of NUP96 proteins even reached 12 nm, which was inconsistent with electron microscope (EM) imaging. Additionally, the Xiao group successfully applied a rhodamine spirolactam (Rh-Gly) probe for PALM application in support of HaloTag (Ye et al., 2019). By incorporating a carboxyl group at a site near the lactam group, newly-synthesized Rh-Gly exhibited enhanced brightness, improved signal-to-noise ratio, desirable temporal resolution (10 s), and excellent accuracy for localization (about 25 nm). Based on the site-specific labeling via HaloTag technique, a super-resolution PALM imaging of HaloTag-fused H2B in Hela and MCF-7 cells was successfully achieved. It is reasonable to predict that more HaloTag-based techniques will be involved in and effectively promote the development of advanced imaging systems.

### In vivo imaging

**FIGURE 5.**
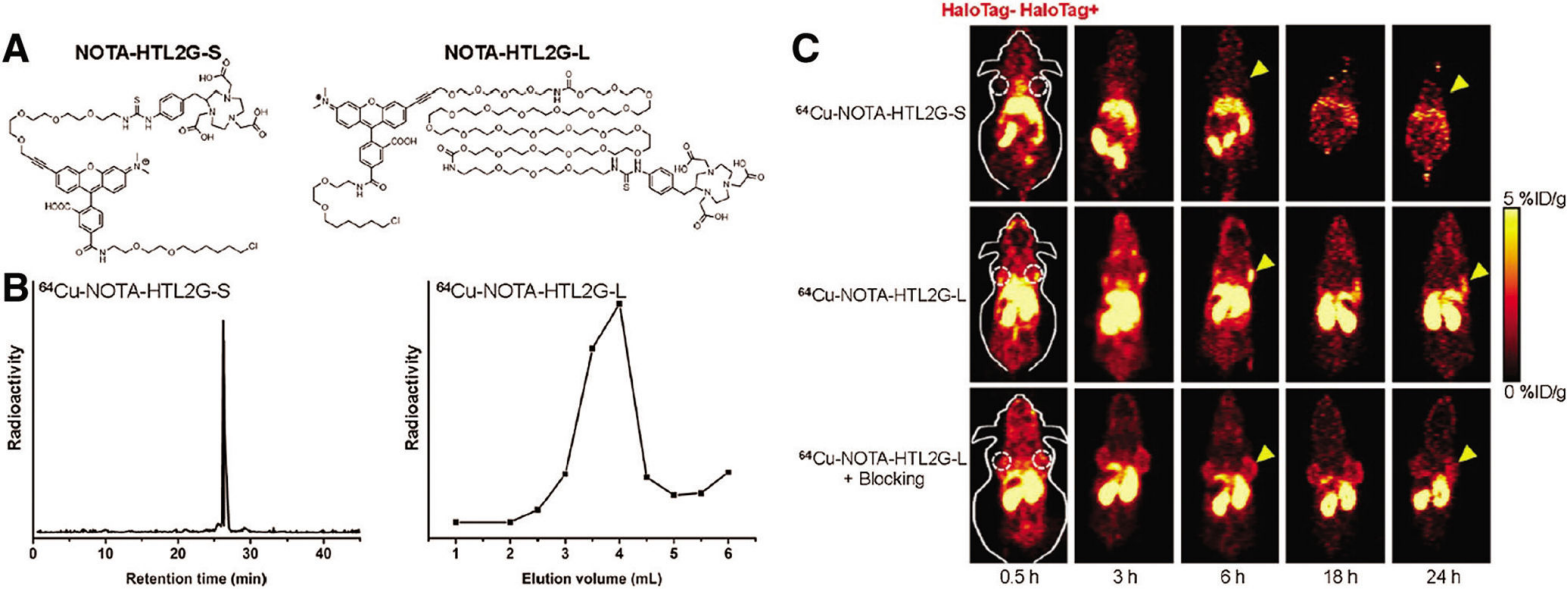
(A) Chemical structures of NOTA-HTL2G-S and NOTA-HTL2G-L. (B) HPLC of ^64^Cu-NOTA-HTL2G-S and size exclusion column chromatography of ^64^Cu-NOTA-HTL2G-L. (C) Serial PET images of mice bearing both 4T1 (left) and 4T1-HaloTag-ECS (right) tumors at different time points post-injection of ^64^Cu-NOTA-HTL2G-S, ^64^Cu-NOTA-HTL2G-L, or ^64^Cu-NOTA-HTL2G-L with blockage (N = 4). Reproduced with permission from Hong et al. (2013).

Due to its high specificity, the HaloTag system demonstrates great potential for various *in vivo* imaging technologies, such as PET, which is highly sensitive, quantitative, and clinically-wide used (Gan et al., 2020; Jadvar et al., 2007; ten Hove et al., 2021). Notably, the Cai group has successfully applied this strategy for various PET-imaging via Cu-64 labeling HTLs (Hong et al., 2011, 2013). More specifically, a string of NOTA-conjugated HTL constructs was synthesized and evaluated for their PET imaging efficiencies (labeled Cu-64) in 4T1-HaloTag-ECS cells or mice bearing 4T1-HaloTag-ECS tumors (Figs. 5A and 5B). Among all, the ligand ^64^Cu-NOTA-HTL2G-L demonstrated the best capability for targeting 4T1-HaloTag-ECS tumor with 4.0 ± 0.2% ID/g at 6 h post injection (Fig. 5C), clearly illustrating the feasibility of the HaloTag system for *in vivo* tracking of a POI or targeting cells. Additionally, the Cornelissen lab further demonstrated the viability of HaloTag in ImmunoPET imaging, reporting HaloTag-labeled PET tracers for tumor pre-targeted ImmunoPET imaging (Knight et al., 2015, 2017).

Furthermore, Knight et al. (2015, 2017) mediated a conjugation of HaloTag to anti-HER2 (Trastuzumab) and TAG-72 (CC49) antibodies via a Lys-to-Lys reaction. SPECT Imaging with ^111^In-HLT-3 showed that tumor uptakes were about 2.8 ± 1.0% ID/g (Trastuzumab) and 3.2 ± 0.3 ID/g (CC49) at 4 h post injection, while internalizations of tracers in the control group were about 2.1 ± 0.4% or 2.1 ± 0.3 ID/g. Notably, these pre-targeted PET imaging techniques offered relatively lower imaging efficiency, which may be caused by the physiochemical features of HTL, especially the limited circulation time.

Given these, Masch et al. (2018) administrated a fluorescent dye conjugated HTL (SiR-HTL) via intracranial injection on the surface of brain to enhance targeting efficiency. High-resolution imaging of the PSD95-HaloTag was achieved via STED (stimulated emission depletion) nanoscopy, with the smallest measured widths (FWHM) being about 50–60 nm. This shows that novel designs of HTLs or tracers with HaloTags would increase the feasibility of HaloTag for translating from basic research to clinical trials.

## Biomolecule Immobilization via HaloTag

### POI purification and collection

Besides, HaloTag system also demonstrates desirable efficiency in the purification and harvest of POI, which has been widely evaluated. The fusion of HaloTag allows POIs to form a covalent bond and be extracted (from bacterial or cell lysis) by the HTL immobilized on solid phase (beads, resin or nano/macroparticles) via a rapid, specifical, and strong binding procedure (Döbber et al., 2018; Friedman Ohana et al., 2016; Liu et al., 2020; Norris et al., 2020; Peschke et al., 2017; Kilpatrick et al., 2017). Notably, this highly efficient HaloTag approach shows a great advantage in enzyme purification instead of chromatographic purification (Erkelenz et al., 2011; Döbber et al., 2018). For instance, two HaloTag fusion enzymes, HaloTag-*Pp*BFD L476Q and HaloTag-*Lb*ADH, were effectively harvested via a simple commercial immobilization procedure (HaloLink™ Resin) in a recent study (Döbber et al., 2018). These enzymes demonstrated desirable abilities for single or cascade biocatalysts, with a high space-time yield of 1850 g/L/d for first-step catalysts and 38 g/L/d for secondstep catalysts. Notably, the immobilized HaloTag-LbADH was still active even two weeks later.

Meanwhile, an advanced system named Serial Capture Affinity Purification (SCAP) was reported by Liu et al. (2020). The interaction between two POI (Spindlin1 and SPINDOC incorporated SNAP-tag or HaloTag, respectively) was systemically examined via Förster resonance energy transfer (FRET), fluorescence cross-correlation spectroscopy quantitative imaging and SCAP cross-linking mass spectrometry (MS). The signal was generated via FRET between TMRDirect™ HTL (HaloTag) and 505-Start SNAP ligand (SNAP) during the interaction. Then, after the efficient enrichment of the protein complex by serial purifications (SNAP and HaloTag capture magnetic beads), this complex was obtained with high quality. In combination with MS, the spindlin1 and SPINDOC complex could be studied comprehensively, suggesting a structure consisting of a ratio of 2:1.

**FIGURE 6.**
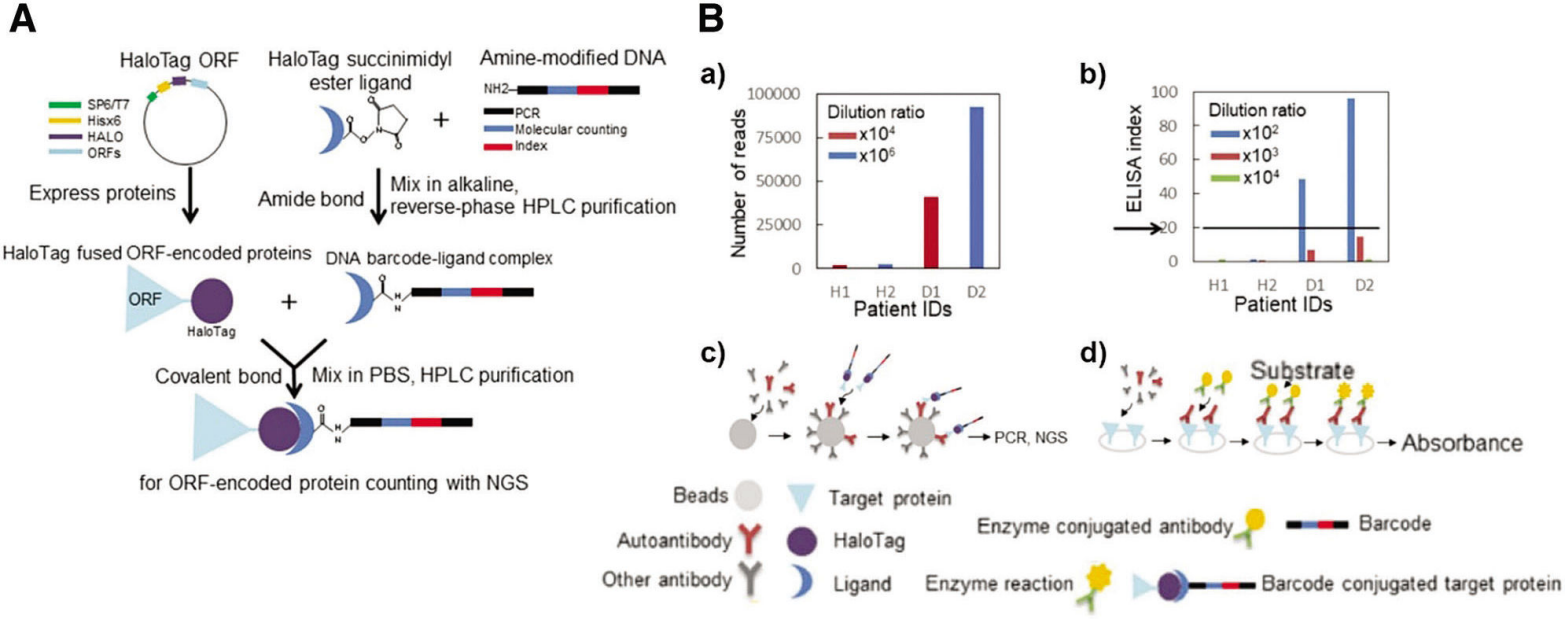
(A) Schematic diagram of the barcoding assay via HaloTag protein. The HTL is linked with specific amino-modified oligonucleotides as DNA barcodes. (B) Autoimmune antibody in patient serum detected via (a,c) barcoding assay or (b,d) conventional ELISA. Reproduced with permission from Yazaki et al. (2020).

Most importantly, this specific-purifying technology also fulfills the high demands of clinical examinations in terms of specificity and quality. Studies were extended to patients’ serum assays for screening autoantibodies, such as p53, GTF2B and Desmoglein 3 (Garranzo-Asensio et al., 2016, 2020; Yazaki et al., 2020). For instance, Barderas group recruited HaloTag fused POI (e.g., p53 and GTF2B) for detecting autoantibodies in serum via ELISA (Garranzo-Asensio et al., 2016, 2020). In a different way, Yazaki et al. (2020) creatively induced a unique DNA single-strand into a POI-HaloTag complex and applied the next-generation sequencing (NGS) as an analytical procedure for serum assays (Fig. 6A). The 1:1 conjugated oligonucleotide and NGS ensure the accuracy of the HaloTag system and dramatically enhance the sensitivity of detection, showing over 10^4^ times wider dynamic range than ELISA (Fig. 6B).

### Investigation of protein and nucleic acid interaction

Numerous interactions between proteins and nucleic acids, ranging from transcription factors & ORF to RNA-binding proteins (RBPs) & RNA, have been involved in most physiological activities (e.g., regulation of gene expression and development). Although these complicated interacting networks are intricate for most technologies, the HaloTag system shows desirable potential in exploring such interactions. After the attachment of a HaloTag, some studies have shown that POI and interacting molecules (e.g., RNA or proteins) could be immobilized for further analysis (Brannan et al., 2016; Li et al., 2020). For example, Brannan et al. (2016) developed an RBP classifying strategy termed SONAR, and subsequently used it to discover new RBPs attaching to the same RNA as a HaloTag fused RBP. With MS and enhanced crosslinking-immunoprecipitation (CLIP), 12 nuclear and cytoplasmic RBPs were successfully identified and investigated. Meanwhile, a Halo-Enhanced Ago2 Pull-Down (HEAP) was designed for identifying micro-RNA (miRNA) targets (Li et al., 2020). Specifically, the harvest and purity of miRNAmRNA-Ago2-HaloTag were greatly ensured by the HaloTag system *in vivo* via CLIP. This strategy was further evaluated and validated in mouse embryonic stem cells (mESCs), developing embryos, and adult tissues. After HaloTagmediated harvest, a strong signal of miR-200bc-3p was observed in 3’UTR *Zeb2*, (EMT regulator) in autochthonous mouse-bearing human brain and lung cancers, which was in agreement with previous studies (Si et al., 2017).

### Protein structure analysis

Additionally, HaloTag anchoring techniques create an excellent opportunity to understand protein structure more intimately. Although the force-clamp atomic force microscope (AFM) has demonstrated its ability to assist in studying protein dynamics, the issue of mechanical drift strongly limits its sensitivity and measuring duration. To remedy this, the Fernández group creatively generated novel magnetic tweezers by combining the HaloTag approach and active correction of the focal drift. The As-designed system monitored protein L folding dynamics at low force (0–60 pN) for up to two weeks, enabling the long-term study of protein folding and misfolding, like chronic traumatic brain damage. Subsequently, Popa *et al.* further generated a mice model carrying a HaloTag-TEV genetic cassette for examining protein dynamics (titin) *in vivo* under various forces (Rivas-Pardo et al., 2020). As expected, the POI, HaloTag-TEV-titin could be specifically severed and immobilized via TEV protease and HTL. Under force generated by magnetic tweezers, Popa et al. (2016) found the titin (I-band region) domains remained in the unfolded state and created 41.5 ZJ of mechanical work for refolding when the puling force was less than 10 pN. It can be expected that this HaloTag-based strategy would promote the exploration of biomechanical functions associated with various proteins.

## Targeted Degradation via HaloTag

Affinity-directed protein missile (AdPROM) system is a functional protein complex that could trigger the degradation of specific proteins. In combination with PROTACs and HaloTag, Buckley et al. (2015) developed a novel AdPROM, named HaloPROTACs. As critical factors, the interaction of HTL and fused HaloTag directly induce the ubiquitylation on POI via the bridging of E3 ubiquitin ligase (Caine et al., 2020; Schiedel et al., 2020; Simpson et al., 2020). Based on HaloPROTACs, Simpson et al. (2020) successfully synthesized a ligand-inducible AdPROM (L-AdPROM) system by combining two site-specific binding strategies, HaloTag and von Hippel-Lindau (VHL). The applied ligand consisted both of HTL and VHL moiety, which could induce the specific binding between VHL-fused AdPROM and FLAG-aGFP_6M_ (anti-GPF nanobody)-Halo. Subsequently, the degradation of FLAG-aGFP_6M_-Halo and its complexes such as FLAG-aGFP_6M_-Halo and GFP-POI could be induced. More importantly, such an L-AdPROM system could mediate an effective degradation (around 50%) of endogenous RAS via FLAG-Halo-aH/KRAS, indicating its potential for degrading any intracellular POI. Similarly, a Cas9 CRISPR-based TRAnscription Factor Targeting Chimeras (TRAFTACs) was recently developed from the haloPROTACs platform (Samarasinghe et al., 2021). A chimeric oligo including transcription factor of interesting (TOI) binding double-stranded DNA (dsDNA) and Cas9 CRISPR-binding RNA (CR-RNA) was prepared first. As a linker, this chimeric oligonucleotide could connect TOI and dCas9-HaloTag7 fusion protein (dCas9HT7). With the attendance of haloPROTAC and VHL-E3 ligase, TRAFTACs could effectively trigger the ubiquitination and proteasomal degradation of oncogenic TOI, such as NF-kB and brachyury. The brachyury targeting TRAFTACs was able to affect the formation of tails in zebrafish, showing a successful implementation of TRAFTACs for *in-vivo* application.

## Other Applications of HaloTag

Notably, biomolecules on the cellular surface play crucial roles in numerous biochemical progress, ranging from immune response to cell differentiation. In addition to cell tracking or immunization via the fusion of HaloTag on the cell membrane, this site-specific binding domain is potentially accessible to a series of functional chemo/biomolecules. Several kinds of research were conducted to study cell development or functional analysis (Pulsipher et al., 2018; Liu et al., 2019). These assays incorporated a HaloTag protein into a cell membrane as surface anchors for further labeling. For example, Liu et al. (2019) labeled HeLa cells with a specific glycopolymer carrying acetylglucosamine and N-acetylmannosamine units, respectively (pMAG or pMAM) via membrane-merged HaloTag, which was able to persist for one week. After incubations with immune cells (e.g., macrophage or dendritic cells), it was observed that binding polymers could efficiently promote M1 marker expression (CD86 and iNOS), dendritic cell maturation and secretion of cytokines (TNF-α and IL-12p70), showing an enhanced anticancer effect. Similarly, Ohno et al. (2014) established a membrane-binding model via HaloTag fusion to investigate the development of stem cell pluripotent embryonic stem cells with attendance of heparan sulfate (HS) glycosaminoglycans (GAGs) (Pulsipher et al., 2018).

## Conclusion and Future Perspectives

Compared with other strategies (e.g., GFP and His-tag), the HaloTag platform offers several advantages for researches requiring high accuracy and specificity. However, there are also several challenges related to the HaloTag system, of which researchers should be particularly attentive. (a) Although the modification will improve its solubility, the structure of HTL (hydrophobic) may still affect its circulation, especially for *in vivo* applications; in addition to innovative designs of HTLs, the pre-loading strategy (e.g., HaloTag-POI labeled with HTL *in vitro* before injection) may act as a potential solution; in contrast, long-term imaging could be also achieved via multiple administration of imaging HTL once high solubility was achieved. (b) Most HTLs used in studies are self-synthesized according to the purpose of their use. In other words, working knowledge and toolset for synthetic chemistry is a basic requirement for using the HaloTag system, which would limit its availability to some extent, such as for some biological labs; for this reason, we suspect that interdisciplinary cooperation may be a trend for future high- quality studies supported by HaloTag technique; (c) As the major feature, HaloTag technique could grant biomolecule with new chemical function via specific labeling and allow this a molecule act as temporal and spatial control. (d) Novel strategies like split HaloTag would promote efficiency and ensure accuracy for monitoring protein and protein interaction (Shao et al., 2021). (e) Given that most studies of HaloTag technology depend on genetic engineering on targeting POI, further applications in clinical trials (which gene-editing could not be easily achieved) are directly hindered. However, the 1:1 ratio labeling capacity greatly ensures the reproduction of complex of HaloTag-fused protein and HTLs, such as PET tracer (i.e., HaloTag fused nanobody and radioactive isotope-labeled HTLs), well meeting the requirements of clinical application. Moreover, such PET tracers could be used for imaging and monitoring the diseases (e.g., tumor) or prognosis in real-time. (f) Besides, changeable HTLs may also offer the potential for the HaloTag-based tracers development for various applications. For instance, the HTLs-ICG conjugation to HaloTag-fused SCFV could be used for imaging guidance surgery. (g) Although the relatively larger size of HaloTag (33 kDa) may be one drawback compared with other more petite tags, like SNAP-tag (19.4 kDa), it may turn into an advantage when it is applied in the integration with SCFV (small-sized protein with short circulation), which may show a better enhancement in the circulation. In this case, extended monitoring could be successfully achieved. Therefore, we believe that the interdisciplinary cooperation and novel HTLs design would greatly promote the future development of HaloTag system, ensuring its efficiency in supporting high accuracy studies (e.g., PET-imaging tracer design).

## Figures and Tables

**TABLE 1:** Comparison of HaloTag among other general tags

Tags	Size (kDa)	Functions	Major applications	Advantages	Drawbacks
His-Tag	0.2–1.6	Affinity	Protein purification	Small size	Non-specific binding
GST-Tag	~26	Affinity/Solubility	Protein purification	High affinity, Improvement of solubility	Large size; Contamination of HSP in mammal cells
Strep-Tags	0.244	Affinity	Protein purification	High affinity	Contamination of biotinylated proteins in mammal cells
EGFP	32.7	Fluorescence	Fluorescence imaging	Brigtness	Large size; Influence of dimer formation
mCherry	28.8	Fluorescence	Fluorescence imaging	Quick formation than other mRFP	Large size
ACP-Tag	9	Site-specific binding or labeling	Imaging of POI	Small size	Impenetrable substrate (surface labeling only)
SNAP/CLIP-Tag	19.4		Targeted studies of POI via imaging, drug delivery and immobilization, etc.	Small size	Relative lower labeling kinetics (2.8 × 10^4^ M^−1^ s^−1^)
HaloTag	33		Targeted studies of POI via imaging, drug delivery, immobilization, structure study and targeted degradation, etc.	Rapid labelling kinetics (2.7 × 10^6^ M^−1^ s^−1^); High stability;	Large size

Note: GST: Glutathione-S-transferase; EGFP: Enhanced green fluorescence protein; HSP: Heat-shock proteins; mRFP: Monomeric red fluorescent protein.

**TABLE 2:** Recent advanced investigations associated with HaloTag technology

Application	HaloTag fused protein	HTL modification	Fusion/testing model	Design of assay	Highlights	Reference
BRET-induced FL-Imaging/drug delivery	Nanoluc	Coumarinibrutinib	SKBR3 cells	Luminescence induced BRET and hyrolysis	Novel design of luciferase Induced BRET and bioluminolysis	(Chang et al., 2019)
FL-Imaging	LC3	AF488p-MILs, TMR-MPLs	U-2 OS cells	HTLs (MILs and MPLs) completion for halotag-LCs labeling	Discover ESCRT machenism of phagophore closure	(Takahashi et al., 2019)
FL-Imaging	5HT_6_	BAPTAJF549 derivatives	hRPE/neuron cells	Ca^2+^ chelation induced FL emission	Develop sensitive indicator for Ca^2+^ detection	(Deo et al., 2019)
FL-Imaging	PDGFR/DAF	PEG-RhoVR	Rat neuron cells	Rho VR induced FL emission	Develop sensor for high speed imaging in brain	(Deal et al., 2020)
FL-Imaging	OM/CM	JF646	*E. coli*	Difference of HTL-labeling area between intact or broken *E. coli*	Evaluate aniti-bacterial effects of various AMP	(Yang and Weisshaar, 2018)
FL-Imaging/drug delivery	Mitochondria	Streptavidin/CPS	Hela cells	4 binding sites on CPS that could carry drug or FL	Develop CPS platform for multiple applications	(López-Andarias et al., 2020)
FL-Imaging	Nup96	Cy5/AF647	U2OS cells	Specific labelling of HTL on nucler pore	Set a reference as SMLM control	(Thevathasan et al., 2019)
SPECT-Imaging	N/A	DTPA-^111^ln	LS174T tumorbearing mice	Labeling of HaloTag & Antibody via click chemistry	Set 2-step SPECT imaging for tumor detection	(Knight et al., 2017)
FL-Imaging	PSD95	SiR	PSD95-HaloTag knocked in mice	Specific labelling of HTL to postsynaptic membrane (PSD95)	*In-vivo* brain (PSD95) STED imaging development	(Masch et al., 2018)
Proteins immobilization	SPIN1	Beads/TMR and 505 start	293FRT cells lysis	Pre-imaging of interaction via FRET; Solid phase immobilization	SNAP & HaloTag twostep purification of bound POI	(Liu et al., 2020)
Protein (enzyme) immobilization	PpBFD, L476Q and LbADH	Beads (Resins)	*E. coli*	Solid phase immobilization via HTL	Effect purification of enzyme (with sustained biocatalyst)	(Döbber et al., 2018)
NGS & Proteins immobilization	Autoantibody (DGS)	DNA barcodes	Human serum	Protein detection via DNA barcode reading (PCR and NGS)	High sensitivity (10^4^ times wider range than traditional ELISA)	(Yazaki et al., 2020)
Protein & miRNA immobilization	Ago2	Beads/TMR	Developing embryos, mESCs, adult tissues	UV mediated protein & miRNA crosslinking; RNA sequencing	Detection of miRNA in mice with/without tumor	(Li et al., 2020)
Magnetic tweezers	Titin-TEV	Glass	HaloTag-TEV titin mice	TEV-based isolation; protein length study under magnetic force	Develop magnetic tweezers for analyzing folding mechanisms	(Rivas-Pardo et al., 2020)

Note: FL: Fluorescence; SPECT: Single-photon emission computerized tomography; ESCRT: Endosomal sorting complex required for transport; NGS: Next-generation sequencing; OM/CM: Outer and cytoplasmic membrane; SiR: Silicon rhodamine; STED: Stimulated emission depletion; SMLM: Single-molecule localization microscopy; POI: Protein of interest; CPS: Cell-penetrating streptavidin; AMPs: Antimicrobial peptides; FRET: Förster resonance energy transfer; PeT: Photo-induced electron transfer.

## References

[R1] Barlag B, Beutel O, Janning D, Czarniak F, Richter CP (2016). Single molecule super-resolution imaging of proteins in living *Salmonella enterica* using self-labelling enzymes. Scientific Reports 6: 1–14. DOI 10.1038/srep31601. 27534893 PMC4989173

[R2] Berki T, Bakunts A, Duret D, Fabre L, Ladavière C, Orsi A, Charreyre MT, Raimondi A, van Anken E, Favier A (2019). Advanced fluorescent polymer probes for the site-specific labeling of proteins in live cells using the HaloTag technology. ACS Omega 4: 12841–12847. DOI 10.1021/acsomega.9b01643. 31460409 PMC6682114

[R3] Brannan KW, Jin W, Huelga SC, Banks CAS, Gilmore JM (2016). SONAR discovers RNA-binding proteins from analysis of large-scale protein-protein interactomes. Molecular Cell 64: 282–293. DOI 10.1016/j.molcel.2016.09.003. 27720645 PMC5074894

[R4] Buckley DL, Raina K, Darricarrere N, Hines J, Gustafson JL, Smith IE, Miah AH, Harling JD, Crews CM (2015). HaloPROTACS: Use of small molecule PROTACs to induce degradation of HaloTag fusion proteins. ACS Chemical Biology 10: 1831–1837. 26070106 10.1021/acschembio.5b00442 PMC4629848

[R5] Butkevich AN, Mitronova GY, Sidenstein SC, Klocke JL, Kamin D (2016). Fluorescent rhodamines and fluorogenic carbopyronines for super-resolution STED microscopy in living cells. Angewandte Chemie International Edition 55: 3290–3294. DOI 10.1002/anie.201511018. 26844929 PMC4770443

[R6] Caine EA, Mahan SD, Johnson RL, Nieman AN, Lam N, Warren CR, Riching KM, Urh M, Daniels DL (2020). Targeted protein degradation phenotypic studies using HaloTag CRISPR/Cas9 endogenous tagging coupled with HaloPROTAC3. Current Protocols in Pharmacology 91: 1–21. DOI 10.1002/cpph.81. PMC7818660 33332748

[R7] Chang D, Lindberg E, Feng S, Angerani S, Riezman H, Winssinger N (2019). Luciferase-induced photouncaging: Bioluminolysis. Angewandte Chemie International Edition 58: 16033–16037. 31478317 10.1002/anie.201907734

[R8] Deal PE, Liu P, Al-Abdullatif SH, Muller VR, Shamardani K, Adesnik H, Miller EW (2020). Covalently tethered rhodamine voltage reporters for high speed functional imaging in brain tissue. Journal of the American Chemical Society 142: 614–622. 31829585 10.1021/jacs.9b12265 PMC6949409

[R9] Deo C, Sheu SH, Seo J, Clapham DE, Lavis LD (2019). Isomeric tuning yields bright and targetable red Ca^2+^ indicators. Journal of the American Chemical Society 141: 13734–13738. 31430138 10.1021/jacs.9b06092

[R10] Döbber J, Gerlach T, Offermann H, Rother D, Pohl M (2018). Closing the gap for efficient immobilization of biocatalysts in continuous processes: HaloTag™ fusion enzymes for a continuous enzymatic cascade towards a vicinal chiral diol. Green Chemistry 20: 544–552.

[R11] Erkelenz M, Kuo CH, Niemeyer CM (2011). DNA-mediated assembly of cytochrome P450 BM3 subdomains. Journal of the American Chemical Society 133: 16111–16118. 21919448 10.1021/ja204993s

[R12] Frei MS, Hoess P, Lampe M, Nijmeijer B, Kueblbeck M (2019). Photoactivation of silicon rhodamines via a light-induced protonation. Nature Communications 10: 8054. DOI 10.1038/s41467-019-12480-3. PMC6783549 31594948

[R13] Friedman Ohana R, Levin S, Wood MG, Zimmerman K, Dart ML (2016). Improved deconvolution of protein targets for bioactive compounds using a palladium cleavable chloroalkane capture tag. ACS Chemical Biology 11: 2608–2617. DOI 10.1021/acschembio.6b00408. 27414062

[R14] Gan R, Rosoman NP, Henshaw DJE, Noble EP, Georgius P, Sommerfeld N (2020). COVID-19 as a viral functional ACE2 deficiency disorder with ACE2 related multi-organ disease. Medical Hypotheses 144: 110024. DOI 10.1016/j.mehy.2020.110024. 32758871 PMC7308773

[R15] Garranzo-Asensio M, Guzmán-Aránguez A, Povedano E, Ruiz-Valdepeñas Montiel V, Poves C (2020). Multiplexed monitoring of a novel autoantibody diagnostic signature of colorectal cancer using HaloTag technology-based electrochemical immunosensing platform. Theranostics 10: 3022–3034. DOI 10.7150/thno.42507. 32194852 PMC7053203

[R16] Garranzo-Asensio M, Guzman-Aranguez A, Poves C, Fernandez-Acenero MJ, Torrente-Rodríguez RM (2016). Toward liquid biopsy: Determination of the humoral immune response in cancer patients using halotag fusion proteinmodified electrochemical bioplatforms. Analytical Chemistry 88: 12339–12345. DOI 10.1021/acs.analchem.6b03526. 28193070

[R17] Gruskos JJ, Zhang G, Buccella D (2016). Visualizing compartmentalized cellular Mg^2+^ on demand with small-molecule fluorescent sensors. Journal of the American Chemical Society 138: 14639–14649. DOI 10.1021/jacs.6b07927. 27750004

[R18] Hipp L, Beer J, Kuchler O, Reisser M, Sinske D, Michaelis J, Gebhardt JCM, Knöll B (2019). Single-molecule imaging of the transcription factor SRF reveals prolonged chromatinbinding kinetics upon cell stimulation. Proceedings of the National Academy of Sciences of the United States of America 116: 880–889. DOI 10.1073/pnas.1812734116. 30598445 PMC6338867

[R19] Hong H, Benink HA, Uyeda HT, Valdovinos HF, Zhang Y, Meisenheimer P, Barnhart TE, Fan F, Cai W (2013). HaloTag as a reporter gene: Positron emission tomography imaging with (64)Cu-labeled second generation HaloTag ligands. American Journal of Translational Research 5: 291–302. 23634240 PMC3633972

[R20] Hong H, Benink HA, Zhang Y, Yang Y, Uyeda HT (2011). Halotag: A novel reporter gene for positron emission tomography. American Journal of Translational Research 3: 392–403. 21904659 PMC3158741

[R21] Huet-Calderwood C, Rivera-Molina F, Iwamoto DV, Kromann EB, Toomre D, Calderwood DA (2017). Novel ecto-tagged integrins reveal their trafficking in live cells. Nature Communications 8: 41. DOI 10.1038/s41467-017-00646-w. PMC5603536 28924207

[R22] Jadvar H, Connolly LP, Fahey FH, Shulkin BL (2007). PET and PET/CT in pediatric oncology. Seminars in Nuclear Medicine 37: 316–331. DOI 10.1053/j.semnuclmed.2007.04.001. 17707239

[R23] Jiang X, Zhang C, Chen J, Choi S, Zhou Y (2019). Quantitative real-time imaging of glutathione with subcellular resolution. Antioxidants and Redox Signaling 30: 1900–1910. DOI 10.1089/ars.2018.7605. 30358421 PMC6486671

[R24] Kilpatrick LE, Friedman-Ohana R, Alcobia DC, Riching K, Peach CJ (2017). Real-time analysis of the binding of fluorescent VEGF165a to VEGFR2 in living cells: Effect of receptor tyrosine kinase inhibitors and fate of internalized agonist-receptor complexes. Biochemical Pharmacology 136: 62–75. DOI 10.1016/j.bcp.2017.04.006. 28392095 PMC5457915

[R25] Knight JC, Mosley M, Stratford MRL, Uyeda HT, Benink HA, Cong M, Fan F, Faulkner S, Cornelissen B (2015). Development of an enzymatic pretargeting strategy for dual-modality imaging. Chemical Communications 51: 4055–4058. DOI 10.1039/C4CC10265G. 25660394

[R26] Knight JC, Mosley M, Uyeda HT, Cong M, Fan F, Faulkner S, Cornelissen B (2017). *In vivo* pretargeted imaging of HER2 and TAG-72 expression using the HaloTag enzyme. Molecular Pharmaceutics 14: 2307–2313. 28505463 10.1021/acs.molpharmaceut.7b00172 PMC5499097

[R27] Lang C, Schulze J, Mendel RR, Hänsch R (2006). HaloTag™: A new versatile reporter gene system in plant cells. Journal of Experimental Botany 57: 2985–2992. 16873446 10.1093/jxb/erl065

[R28] Lee HLD, Lord SJ, Iwanaga S, Zhan K, Xie H (2010). Superresolution imaging of targeted proteins in fixed and living cells using photoactivatable organic fluorophores. Journal of the American Chemical Society 132: 15099–15101. 20936809 10.1021/ja1044192 PMC2972741

[R29] Lepore A, Taylor H, Landgraf D, Okumus B, Jaramillo-Riveri S, McLaren L, Bakshi S, Paulsson J, Karoui MEl (2019). Quantification of very low-abundant proteins in bacteria using the HaloTag and epi-fluorescence microscopy. Scientific Reports 9: 1–9. 31133640 10.1038/s41598-019-44278-0 PMC6536506

[R30] Lesiak L, Zhou X, Fang Y, Zhao J, Beck JR, Stains CI (2020). Imaging GPCR internalization using near-infrared Nebraska redbased reagents. Organic & Biomolecular Chemistry 18: 2459. 32167123 10.1039/d0ob00043d PMC7261517

[R31] Li X, Pritykin Y, Concepcion CP, Lu Y, La Rocca G (2020). High-resolution *in vivo* identification of miRNA targets by Halo-Enhanced Ago2 pull-down. Molecular Cell 79: 167–179.e11. 32497496 10.1016/j.molcel.2020.05.009 PMC7446397

[R32] Liu AA, Zhang Z, Sun EZ, Zheng Z, Zhang ZL, Hu Q, Wang H, Pang DW (2016). Simultaneous visualization of parental and progeny viruses by a capsid-specific HaloTag labeling strategy. ACS Nano 10: 1147–1155. DOI 10.1021/acsnano.5b06438. 26720596

[R33] Liu DS, Phipps WS, Loh KH, Howarth M, Ting AY (2012). Quantum dot targeting with lipoic acid ligase and HaloTag for single-molecule imaging on living cells. ACS Nano 6: 11080–11087. DOI 10.1021/nn304793z. 23181687 PMC3528850

[R34] Liu Q, Jiang S, Liu B, Yu Y, Zhao ZA, Wang C, Liu Z, Chen G, Chen H (2019). Take immune cells back on track: Glycopolymerengineered tumor cells for triggering immune response. ACS Macro Letters 8: 337–344. DOI 10.1021/acsmacrolett.9b00046. 35651134

[R35] Liu X, Zhang Y, Wen Z, Hao Y, Banks CAS (2020). Driving integrative structural modeling with serial capture affinity purification. Proceedings of the National Academy of Sciences of the United States of America 117: 31861–31870. DOI 10.1073/pnas.2007931117. 33257578 PMC7749342

[R36] Löchte S, Waichman S, Beutel O, You C, Piehler J (2014). Live cell micropatterning reveals the dynamics of signaling complexes at the plasma membrane. Journal of Cell Biology 207: 407–418. DOI 10.1083/jcb.201406032. 25385185 PMC4226739

[R37] López-Andarias J, Saarbach J, Moreau D, Cheng Y, Derivery E, Laurent Q, González-Gaitán M, Winssinger N, Sakai N, Matile S (2020). Cell-penetrating streptavidin: A general tool for bifunctional delivery with spatiotemporal control, mediated by transport systems such as adaptive benzopolysulfane networks. Journal of the American Chemical Society 142: 4784–4792. DOI 10.1021/jacs.9b13621. 32109058 PMC7307903

[R38] Los GV, Darzins A, Karassina N, Zimprich C, Learish R (2005). HaloTag interchangeable labeling technology for cell imaging and protein capture. Promega Cell Notes 11: 2–6.

[R39] Masch JM, Steffens H, Fischer J, Engelhardt J, Hubrich J (2018). Robust nanoscopy of a synaptic protein in living mice by organic-fluorophore labeling. Proceedings of the National Academy of Sciences of the United States of America 115: E8047–E8056. DOI 10.1073/pnas.1807104115. 30082388 PMC6112726

[R40] Matsui Y, Funato Y, Imamura H, Miki H, Mizukami S, Kikuchi K (2017). Visualization of long-term Mg^2+^ dynamics in apoptotic cells using a novel targetable fluorescent probe. Chemical Science 8: 8255–8264. DOI 10.1039/C7SC03954A. 29619172 PMC5858021

[R41] Naested H, Fennema M, Hao L, Andersen M, Janssen DB, Mundy J (1999). A bacterial haloalkane dehalogenase gene as a negative selectable marker in Arabidopsis. Plant Journal 18: 571–576. DOI 10.1046/j.1365-313X.1999.00477.x. 10417708

[R42] Neklesa TK, Tae HS, Schneekloth AR, Stulberg MJ, Corson TW, Sundberg TB, Raina K, Holley SA, Crews CM (2011). Small-molecule hydrophobic tagging-induced degradation of HaloTag fusion proteins. Nature Chemical Biology 7: 538–543. DOI 10.1038/nchembio.597. 21725302 PMC3139752

[R43] Norris JL, Patel T, Dasari AKR, Cope TA, Lim KH, Hughes RM (2020). Covalent and non-covalent strategies for the immobilization of Tobacco Etch Virus protease (TEVp) on superparamagnetic nanoparticles. Journal of Biotechnology 322: 1–9. DOI 10.1016/j.jbiotec.2020.06.021. 32619644

[R44] Ohno M, Karagiannis P, Taniguchi Y (2014). Protein expression analyses at the single cell level. Molecules 19: 13932–13947. 25197931 10.3390/molecules190913932 PMC6270791

[R45] Parvez S, Long MJC, Lin HY, Zhao Y, Haegele JA, Pham VN, Lee DK, Aye Y (2016). T-REX on-demand redox targeting in live cells. Nature Protocols 11: 2328–2356. DOI 10.1038/nprot.2016.114. 27809314 PMC5260244

[R46] Peach CJ, Kilpatrick LE, Woolard J, Hill SJ (2021). Use of NanoBiT and NanoBRET to monitor fluorescent VEGF-A binding kinetics to VEGFR2/NRP1 heteromeric complexes in living cells. British Journal of Pharmacology 178: 2393–2411. DOI 10.1111/bph.15426. 33655497

[R47] Peraro L, Deprey KL, Moser MK, Zou Z, Ball HL, Levine B, Kritzer JA (2018). Cell penetration profiling using the chloroalkane penetration assay. Journal of the American Chemical Society 140: 11360–11369. DOI 10.1021/jacs.8b06144. 30118219 PMC6205923

[R48] Peschke T, Rabe KS, Niemeyer CM (2017). Orthogonal surface tags for whole-cell biocatalysis. Angewandte Chemie International Edition 56: 2183–2186. DOI 10.1002/anie.201609590. 28105787

[R49] Popa I, Rivas-Pardo JA, Eckels EC, Echelman DJ, Badilla CL, Valle-Orero J, Fernández JM (2016). A HaloTag anchored ruler for week-long studies of protein dynamics. Journal of the American Chemical Society 138: 10546–10553. DOI 10.1021/jacs.6b05429. 27409974 PMC5510598

[R50] Pulsipher A, Griffin ME, Stone SE, Hsieh-Wilson LC (2018). Long-lived glycan engineering to direct stem cell fate. Physiology & Behavior 176: 139–148.

[R51] Raina K, Noblin DJ, Serebrenik YV, Adams A, Zhao C, Crews CM (2014). Targeted protein destabilization reveals an estrogenmediated ER stress response. Nature Chemical Biology 10: 957–962. DOI 10.1038/nchembio.1638. 25242550 PMC4324732

[R52] Rivas-Pardo JA, Li Y, Mártonfalvi Z, Tapia-Rojo R, Unger A (2020). A HaloTag-TEV genetic cassette for mechanical phenotyping of proteins from tissues. Nature Communications 11: 1–13. DOI 10.1038/s41467-020-15465-9. PMC7189229 32345978

[R53] Samarasinghe KTG, Jaime-Figueroa S, Burgess M, Nalawansha DA, Dai K, Hu Z, Bebenek A, Holley SA, Crews CM (2021). Targeted degradation of transcription factors by TRAFTACs: TRAnscription Factor TArgeting Chimeras. Cell Chemical Biology 28: 648–661.e5. DOI 10.1016/j.chembiol.2021.03.011. 33836141 PMC8524358

[R54] Samelson AJ, Bolin E, Costello SM, Sharma AK, O’brien EP (2018). Kinetic and structural comparison of a protein’s cotranslational folding and refolding pathways. Science Advances 4: eaas9098. http://advances.sciencemag.org/. 29854950 10.1126/sciadv.aas9098 PMC5976279

[R55] Sato R, Kozuka J, Ueda M, Mishima R, Kumagai Y, Yoshimura A, Minoshima M, Mizukami S, Kikuchi K (2017). Intracellular protein-labeling probes for multicolor single-molecule imaging of immune receptor-adaptor molecular dynamics. Journal of the American Chemical Society 139: 17397–17404. DOI 10.1021/jacs.7b08262. 29119782

[R56] Schiedel M, Lehotzky A, Szunyogh S, Oláh J, Hammelmann S (2020). HaloTag-targeted sirtuin-rearranging ligand (SirReal) for the development of proteolysis-targeting chimeras (PROTACs) against the lysine deacetylase sirtuin 2 (Sirt2)**. ChemBioChem 21: 3371–3376. DOI 10.1002/cbic.202000351. 32672888 PMC7754454

[R57] Schlichthaerle T, Strauss MT, Schueder F, Auer A, Nijmeijer B (2019). Direct visualization of single nuclear pore complex proteins using genetically-encoded probes for DNA-PAINT. Angewandte Chemie International Edition 58: 13004–13008. DOI 10.1002/anie.201905685. 31314157 PMC6771475

[R58] Shao S, Zhang H, Zeng Y, Li Y, Sun C, Sun Y (2021). TagBiFC technique allows long-term single-molecule tracking of protein-protein interactions in living cells. Communications Biology 4: 19. DOI 10.1038/s42003-021-01896-7. 33742089 PMC7979928

[R59] Si L, Tian H, Yue W, Li L, Li S, Gao C, Qi L (2017). Potential use of microRNA-200c as a prognostic marker in non-small cell lung cancer. Oncology Letters 14: 4325–4330. DOI 10.3892/ol.2017.6667. 28943946 PMC5604169

[R60] Simpson LM, Macartney TJ, Nardin A, Fulcher LJ, Röth S, Testa A, Maniaci C, Ciulli A, Ganley IG, Sapkota GP (2020). Inducible degradation of target proteins through a tractable affinity-directed protein missile system. Cell Chemical Biology 27: 1164–1180.e5. DOI 10.1016/j.chembiol.2020.06.013. 32668203 PMC7505680

[R61] Spencer AC, Singh V, Mendoza DV, Margolin W, Kool ET (2018). Light-up channel dyes for haloalkane-based protein labeling *in vitro* and in bacterial cells. Physiology & Behavior 176: 139–148. 10.1021/acs.bioconjchem.6b00613 PMC5572799 27998074

[R62] Strakova K, Lopez-Andarias J, Jimenez-Rojo N, Chambers JE, Marciniak SJ, Riezman H, Sakai N, Matile S (2020). Haloflippers: A general tool for the fluorescence imaging of precisely localized membrane tension changes in living cells. ACS Central Science 6: 1376–1385. DOI 10.1021/acscentsci.0c00666. 32875078 PMC7453570

[R63] Strauch RC, Mastarone DJ, Sukerkar PA, Song Y, Ipsaro JJ, Meade TJ (2011). Reporter protein-targeted probes for magnetic resonance imaging. Journal of the American Chemical Society 133: 16346–16349. DOI 10.1021/ja206134b. 21942425 PMC3203639

[R64] Taguchi R, Terai T, Ueno T, Komatsu T, Hanaoka K, Urano Y (2018). A protein-coupled fluorescent probe for organelle-specific imaging of Na^+^. Sensors and Actuators B: Chemical 265: 575–581. DOI 10.1016/j.snb.2018.03.090.

[R65] Takahashi Y, He H, Tang Z, Hattori T, Liu Y (2018). An autophagy assay reveals the ESCRT-III component CHMP2A as a regulator of phagophore closure. Nature Communications 9: 207. DOI 10.1038/s41467-018-05254-w. PMC6054611 30030437

[R66] Takahashi Y, Liang X, Hattori T, Tang Z, He H (2019). VPS37A directs ESCRT recruitment for phagophore closure. Journal of Cell Biology 218: 3336–3354. DOI 10.1083/jcb.201902170. 31519728 PMC6781443

[R67] ten Hove D, Treglia G, Slart RHJA, Damman K, Wouthuyzen-Bakker M (2021). The value of 18F-FDG PET/CT for the diagnosis of device-related infections in patients with a left ventricular assist device: A systematic review and metaanalysis. European Journal of Nuclear Medicine and Molecular Imaging. 48: 241–253. Berlin: Springer Science and Business Media Deutschland GmbH. 32594196 10.1007/s00259-020-04930-8 PMC7835315

[R68] Thevathasan JV, Kahnwald M, Cieśliński K, Hoess P, Peneti SK (2019). Nuclear pores as versatile reference standards for quantitative superresolution microscopy. Nature Methods 16: 1045–1053. DOI 10.1038/s41592-019-0574-9. 31562488 PMC6768092

[R69] Yang Z, Weisshaar JC (2018). HaloTag assay suggests common mechanism of *E. coli* membrane permeabilization induced by cationic peptides. Physiology & Behavior 63: 1–18. DOI 10.1021/acschembio.8b00336.Halo. PMC6097915 29812894

[R70] Yazaki J, Kawashima Y, Ogawa T, Kobayashi A, Okoshi M (2020). HaloTag-based conjugation of proteins to barcoding-oligonucleotides. Nucleic Acids Research 48: 2–13. DOI 10.1093/nar/gkz1086. PMC6954424 31752022

[R71] Ye Z, Yu H, Yang W, Zheng Y, Li N (2019). Strategy to lengthen the on-time of photochromic rhodamine spirolactam for super-resolution photoactivated localization microscopy. Journal of the American Chemical Society 141: 6527–6536. DOI 10.1021/jacs.8b11369. 30938994

[R72] Yoon YJ, Wu B, Buxbaum AR, Das S, Tsai A, English BP, Grimm JB, Lavis LD, Singer RH (2016). Glutamate-induced RNA localization and translation in neurons. Proceedings of the National Academy of Sciences of the United States of America 113: E6877–E6886. DOI 10.1073/pnas.1614267113. 27791158 PMC5098659

[R73] Zastrow ML, Huang Z, Lippard SJ (2020). HaloTag-based hybrid targetable and ratiometric sensors for intracellular zinc. ACS Chemical Biology 15: 396–406. DOI 10.1021/acschembio.9b00872. 31917534

[R74] Zhang Y, So MK, Loening AM, Yao H, Gambhir SS, Rao J (2006). HaloTag protein-mediated site-specific conjugation of bioluminescent proteins to quantum dots. Angewandte Chemie International Edition 45: 4936–4940. DOI 10.1002/(ISSN)1521-3773. 16807952

